# Epidemiological and Clinical Characteristics of Facial Bone Fractures across Age Groups: A Single-Center Retrospective Analysis of 4,975 Patients at a Regional Trauma Center in Gangwon Province, South Korea

**DOI:** 10.1055/a-2870-8157

**Published:** 2026-08-11

**Authors:** Woosang Jeon, Minwoo Park, Sug Won Kim, Jiye Kim

**Affiliations:** Department of Plastic and Reconstructive Surgery 65447 Wonju Severance Christian HospitalWonju-siGanwon-doKorea (the Republic of)

**Keywords:** fracture-nasal bone, fractures-mandible, fractures-orbit and zygoma and others, epidemiology, zygomaticomaxillary complex, orbital fracture, elderly, hospitalization

## Abstract

**Background:**

Patient age influences diagnosis, operative planning, and recovery in facial fracture care. We aimed to delineate age-dependent patterns in fracture sites, injury mechanisms, and management across pediatric, working-age, and elderly cohorts at a regional trauma center.

**Methods:**

We retrospectively analyzed 4,975 patients with computed tomography (CT)-confirmed facial fractures (2016–2021), stratified into <20, 20–64, and ≥65 years. Fracture sites, mechanisms, and management (conservative, closed reduction, open reduction and internal fixation [ORIF]) were recorded. Group differences were tested using chi-square/analysis of variance (ANOVA) with Holm adjustment. Multivariable logistic regression identified independent predictors of operative management.

**Results:**

The cohort was predominantly male (∼3:1). Nasal fractures were most common (42.3%) but declined with age (60.1%, 41.7%, 31.5%, respectively). Zygomaticomaxillary complex (ZMC) and orbital fractures increased with age (ZMC: 10.3%, 20.7%, 30.8%, respectively; orbital: 14.2%, 25.6%, 28.1%, respectively). Mandibular fractures were more frequent in younger patients (11.6% vs. 5.7% in patients aged ≥65 years). In the elderly, falls (slip/roll/fall-down combined 52.6%) predominated, whereas assault and sports were proportionally higher in younger groups. Mechanism was strongly associated with fracture site ( *p* < 0.001). Operative treatment was less frequent in the elderly, in whom conservative care predominated. In multivariable analysis, increasing age independently predicted lower odds of surgery ( *p* < 0.001), whereas multiple fractures were the strongest predictor of operative intervention (adjusted odds ratio = 2.50, *p* < 0.001).

**Conclusions:**

Facial fracture patterns, mechanisms, and treatment differ by age. Findings support age-tailored prevention—fall prevention for older adults and high-energy injury mitigation for younger cohorts—and individualized strategies balancing functional priorities against systemic risk in the elderly.

## Introduction

Facial bone fractures are among the most common injuries encountered in trauma patients and often result in functional and aesthetic sequelae. 1 2 The incidence and pattern of these fractures vary according to demographic and socioeconomic factors, as well as the mechanism of injury. 3 4 5 With the demographic transition toward an aging society, the number of elderly patients sustaining facial fractures has been steadily increasing. 1 4 6 Elderly individuals exhibit distinct trauma characteristics due to reduced bone density, slower protective reflexes, and an increased likelihood of low-energy falls. 4 7 8 These age-related differences in host factors and injury mechanisms are expected to influence fracture patterns, treatment selection, and clinical outcomes in everyday practice. Our institution is a tertiary referral hospital and designated regional trauma center for Gangwon Province, functioning as a major hub for facial trauma care. As such, it manages a broad spectrum of facial bone fractures across the lifespan, from pediatric to elderly patients, including both high-energy trauma and low-energy fall injuries. This setting provides an opportunity to assess real-world age-dependent patterns within a large, unselected cohort of patients with computed tomography (CT)-confirmed facial fractures. Understanding age-specific characteristics of facial trauma is crucial for optimizing triage, operative planning, perioperative risk stratification, and targeted prevention strategies. 9 10 11 12 Therefore, this study analyzed the epidemiologic and clinical characteristics of facial bone fractures across three age strata (<20, 20–64, and ≥65 years) treated at our regional trauma center, with emphasis on age-related differences in fracture patterns, etiologies, and treatment modalities.

## Methods

### Study Design and Population

**Fig. 1:**
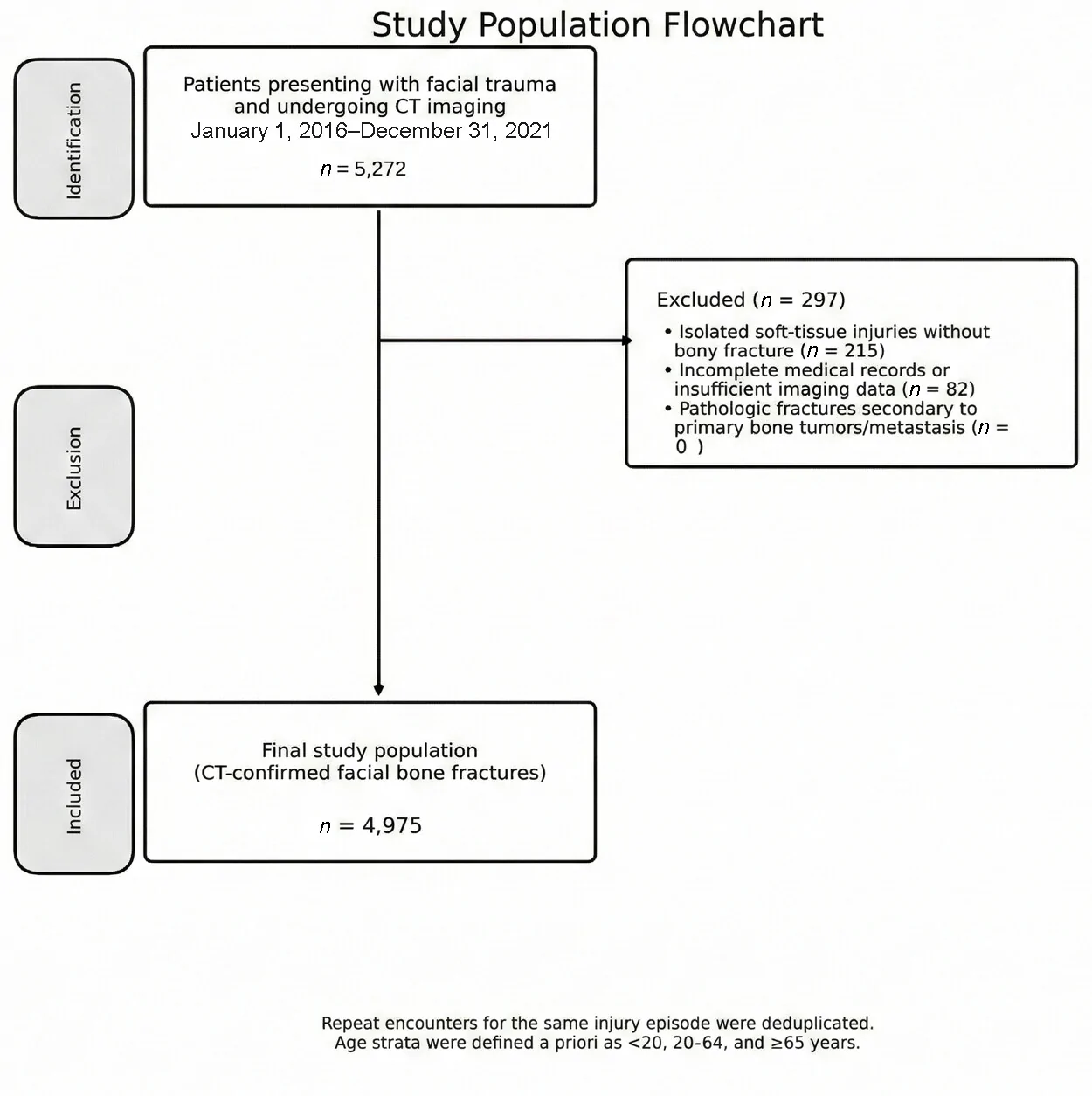
Flow diagram of patient selection for the retrospective cohort study. From January 1, 2016, to December 31, 2021, 5,272 patients presenting with facial trauma and undergoing CT imaging were screened. After excluding 309 cases—isolated soft tissue injuries without bony fracture ( *n* = 215), incomplete medical records or insufficient imaging data ( *n* = 82), and pathologic fractures secondary to primary bone tumors or metastasis ( *n* = 0)—a total of 4,975 patients with CT-confirmed facial bone fractures were included in the final analysis. Repeat encounters within the same injury episode were deduplicated. Age strata were defined a priori as <20, 20–64, and ≥65 years. CT, computed tomography.

This was a single-center retrospective cohort study conducted at a tertiary referral hospital and designated regional trauma center in Gangwon Province, South Korea, covering the period from January 1, 2016, to December 31, 2021. We initially screened 5,272 patients who presented with facial trauma and underwent CT imaging. From this initial cohort, we applied the following exclusion criteria: (1) Isolated soft tissue injuries without evidence of bony fracture ( *n* = 215); (2) incomplete medical records or insufficient imaging data for accurate classification ( *n* = 82); and (3) pathologic fractures secondary to primary bone tumors or metastasis ( *n* = 0). After excluding these 309 cases, a total of 4,975 patients with CT-confirmed facial bone fractures were included in the final analysis. The patient selection process is summarized in Fig. 1 . Repeat encounters for the same injury episode were deduplicated. Age groups were defined a priori as <20 years, 20–64 years, and ≥65 years.

### Variables and Data Collection

We abstracted demographics (age, sex) and comorbidities (including osteoporosis, cognitive impairment, and other conditions that may influence bone fragility or surgical decision-making). Comorbidities were defined based on documented clinical diagnoses in the electronic medical record at the time of admission. Injury–mechanism categories were traffic accident (TA), industrial accident, assault (interpersonal violence), sports, slip-down, roll-down, fall-down, and others/unknown. Fracture sites were initially coded by anatomical region as orbital, zygomaticomaxillary complex (ZMC), maxilla/Le Fort, mandible, nasal, frontal sinus, naso-orbital-ethmoid (NOE), and panfacial. Panfacial fractures were defined as concomitant fractures involving the upper, middle, and lower thirds of the facial skeleton. 13 For the primary analyses, maxillary/Le Fort fractures were merged into the ZMC category. Additionally, orbital wall fractures (floor, medial, lateral, or roof) were grouped into a single “orbital” category, and detailed Le Fort subtypes (I–III) or ZMC fracture severity (e.g., arch vs. body) were not further stratified. Multiple fractures were defined as the simultaneous presence of fractures in two or more distinct anatomical regions (e.g., concomitant nasal and ZMC fractures) within a single injury episode. Management modality was categorized as conservative treatment, closed reduction, or open reduction and internal fixation (ORIF). For regression analyses, management was dichotomized as operative management (closed reduction or ORIF) versus conservative treatment, coded as yes (= 1) versus no (= 0). Timelines included injury-to-surgery interval (days) and length of hospital stay (days) extracted from electronic medical records. All fracture sites were confirmed by review of CT images and radiology reports; CT images were additionally reviewed by two plastic surgeons to verify fracture-site categorization, with discrepancies resolved by consensus.

### Statistical Analysis

Continuous variables were summarized as mean ± standard deviation (SD) or median (interquartile range [IQR]) based on distribution (Shapiro–Wilk test). Categorical variables were summarized as *n* (percentage). Between-group differences used chi-square tests (or Fisher's exact test when expected cell counts <5) for categorical data and analysis of variance (ANOVA or Kruskal–Wallis for non-normal data) for continuous data. When overall tests were significant, pairwise comparisons used Bonferroni (ANOVA) or Dunn's tests with Holm adjustment (Kruskal–Wallis). Where appropriate, effect sizes were reported (Cramér's V for chi-square; eta-squared for ANOVA; epsilon-squared for Kruskal–Wallis). Temporal distributions (day of week, day-of-month bins, and year) were assessed using chi-square tests with Holm-adjusted pairwise comparisons when appropriate. Annual case counts between the pre-COVID-19 and COVID-19 periods were compared using a Poisson rate ratio test, and rate ratios with 95% confidence intervals (CIs) were reported. Two-sided *p* < 0.05 denoted statistical significance. Multivariable logistic regression was performed to identify independent predictors of operative management. Covariates included age, sex, osteoporosis, cognitive impairment, and multiple fractures. Adjusted odds ratios (aORs) with 95% CIs are reported. Missing covariate data were handled using complete-case analysis ( *n* = 4,767). Analyses were performed in R (v4.x) or SPSS (v26+).

## Results

A total of 4,975 patients with facial bone fractures were analyzed. The cohort was predominantly male (male-to-female ratio ∼3:1).

Patients aged 20–64 years comprised the largest proportion, followed by elderly and pediatric patients ( Table 1 ). When we specifically examined comorbidities that were considered potentially related to the prevalence of facial bone fractures in elderly patients—such as cognitive impairment, osteoporosis, and a history of cerebral infarction—comorbidities related to frailty (cognitive impairment, osteoporosis, and prior cerebral infarction) were significantly more prevalent in the elderly group ( Table 1 ).

**Table 1:** General characteristics of the study population ( *n* = 4,975)

Variable	Pediatric (age <20 years)		Adult (age 20–64 years)		Elderly (age ≥65 years)		*p* -Value
	*N*	Percentage	*N*	Percentage	*N*	Percentage	
	621	12.5	3,515	70.7	839	16.9	
Age, years							
Mean ± SD	14.21 ± 4.54	–	43.15 ± 13.44	–	74.3 ± 6.97	–	–
Range	0–19		20–64		65–97		
Sex, number							<0.001
Male	497	80	2,778	79	564	67.2	
Female	124	20	737	21	275	32.8	
Cognitive impairment							<0.001
Yes	2	0.3	38	1.1	34	4.1	
No	619	99.7	3,477	98.9	805	95.9	
Osteoporosis							<0.001
Yes	0	0	4	0.1	9	1.1	
No	621	100	3,511	99.9	830	98.9	
Psychosis							0.927
Yes	6	1	40	1.1	9	1.1	
No	615	99	3,475	98.9	830	98.9	
Cerebral infarction							0.604
Yes	0	0	7	0.2	34	4.1	
No	621	100	3,508	99.8	805	95.9	
Site							0.252
Outdoor	482	77.6	2,678	76.2	657	78.3	
Indoor	139	22.4	837	23.8	182	21.7	
Fracture multiplicity							0.431
Single	604	97.3	3,391	96.5	815	97.1	
Multiple	17	2.7	124	3.5	24	2.9	
Operation							<0.001 b
Yes	354	57	1,596	45.4	215	25.6	
No	267	43	1,919	54.6	624	74.4	

Abbreviations: SD, standard deviation.

Note: Multiple fractures were identified at the case level when two or more distinct fracture-site regions were recorded within a single injury episode. These 165 cases correspond to 81 unique patients (1.7%) with multiple fractures.

Chi-square test was used.

a Significant at 95% confidence level.

b Fisher's exact test was used.

**Fig. 2:**
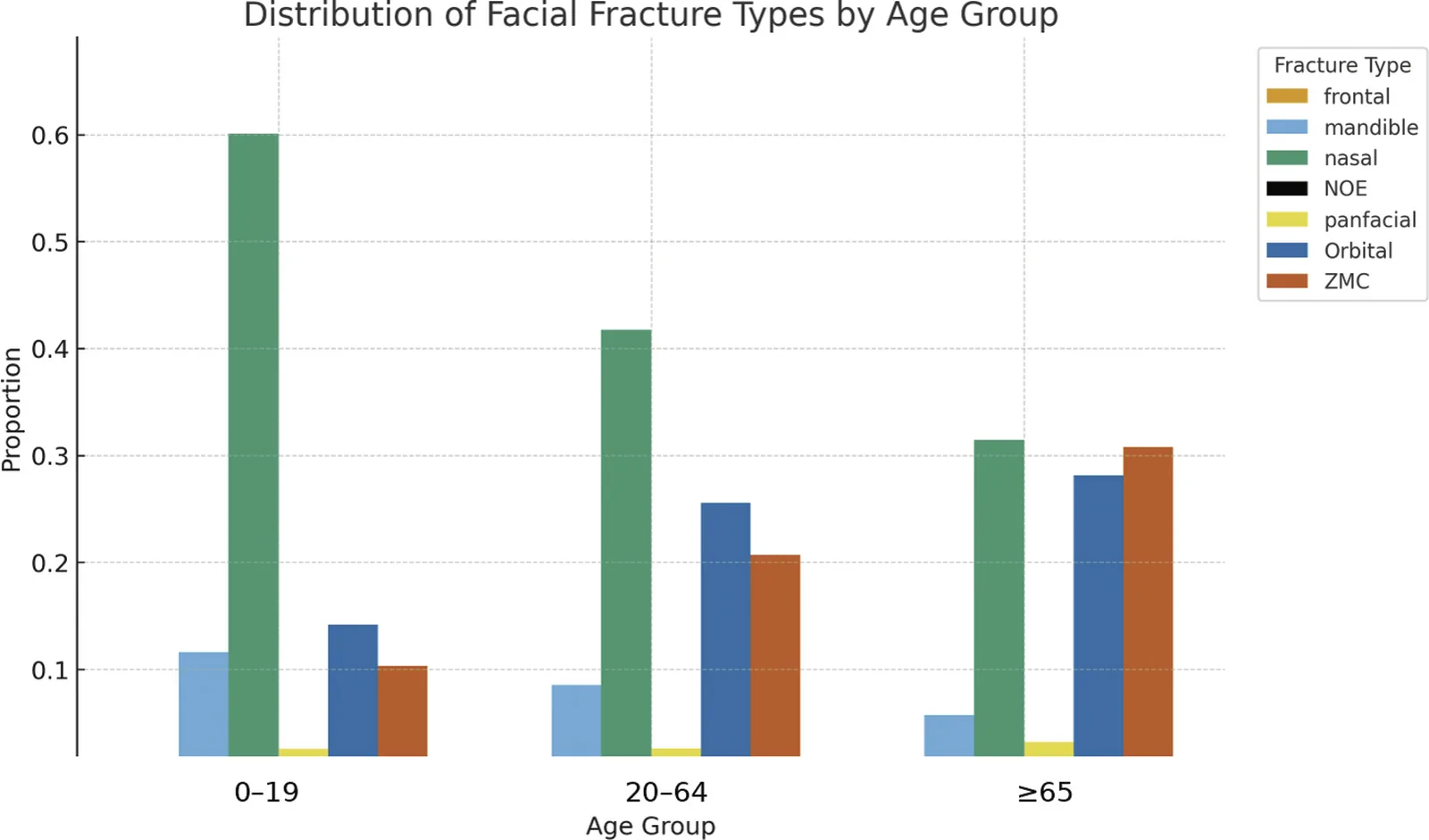
Distribution of facial bone fracture sites by age groups. Nasal bone fractures were the most common across all age groups, but their relative proportion decreased with age. Zygomaticomaxillary complex (ZMC) and orbital fractures became relatively more prevalent in the elderly, while mandibular fractures predominated in younger patients. NOE, naso-orbito-ethmoidal.

**Fig. 3:**
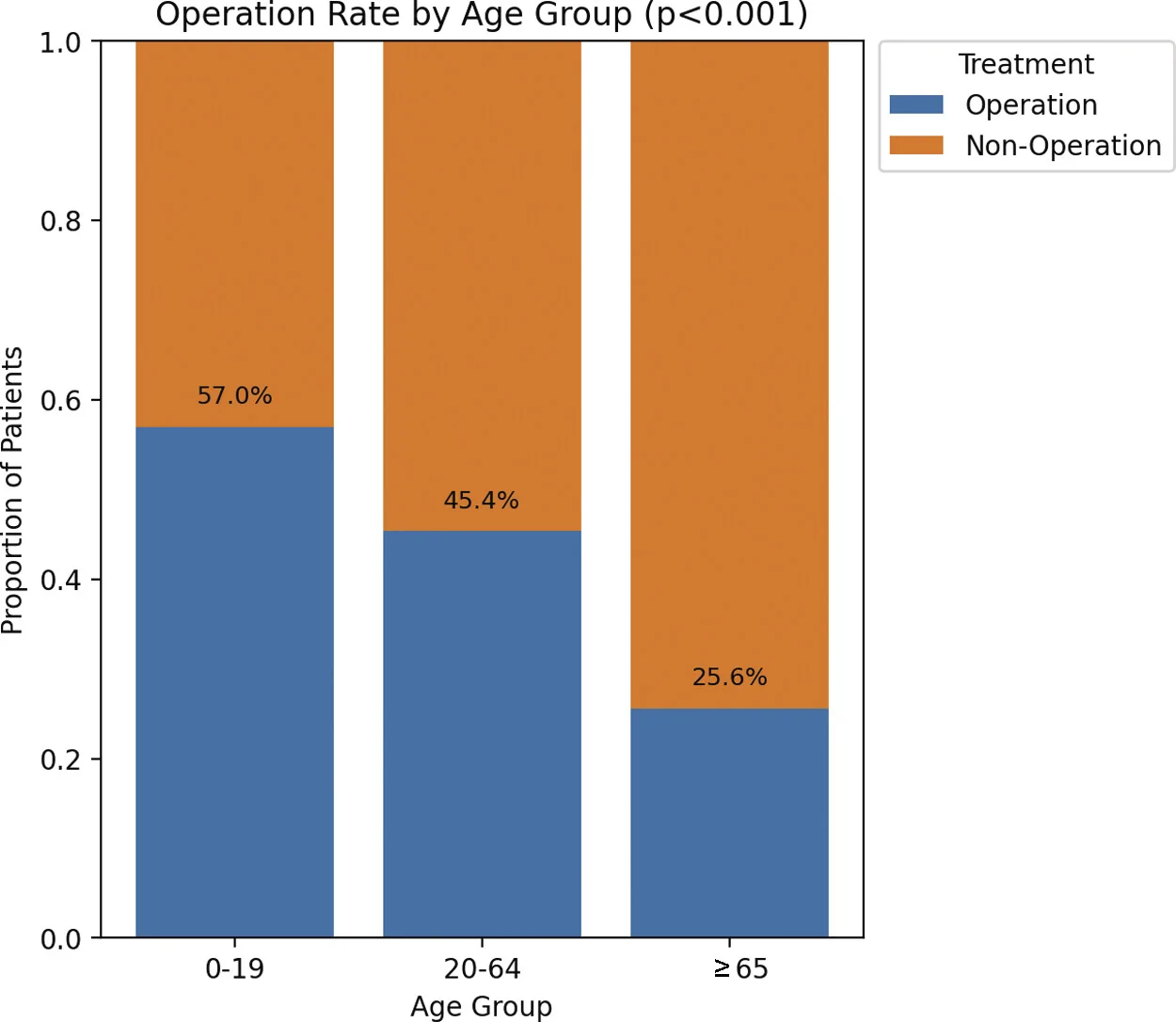
Treatment modalities by age group. Operative management (closed reduction or ORIF) was most frequent in the pediatric group (57.0%) and least frequent in the elderly group (25.6%; *p* < 0.001). ORIF, open reduction and internal fixation.

Fracture type distribution differed significantly across age groups ( *p* < 0.001). Nasal bone fractures were most common overall ( *n* = 2,104, 42.3%), but their relative proportion declined with increasing age (<20, 20–64, and ≥65 years: 60.1%, 41.7%, and 31.5%, respectively). In contrast, the proportions of ZMC and orbital fractures were higher among elderly patients (ZMC: 10.3%, 20.7%, and 30.8%; orbital: 14.2%, 25.6%, and 28.1% in the <20, 20–64, and ≥65-year groups, respectively). Mandibular fractures showed the opposite pattern, being more frequent in younger patients (11.6% in <20 years vs. 5.7% in ≥65 years), paralleling greater exposure to high-energy trauma such as TAs and assaults ( Table 2 and Fig. 2 ). Management strategies also varied by age ( *p* < 0.001): Surgical treatment was most frequent in younger patients, whereas conservative management was more common in elderly patients, which may reflect age-related frailty and perioperative risk considerations not fully captured by recorded comorbidities ( Fig. 3 ).

**Table 2:** Facial bone fracture type ( *n* = 4,975)

Variable	Pediatric (age <20 years)		Adult (age 20–64 years)		Elderly (age ≥65 years)		*p* -Value
	*N*	Percentage	*N*	Percentage	*N*	Percentage	
	621	12.5	3,515	70.7	839	16.9	
Frontal							0.187 ^a^
Yes	8	1.3	25	0.7	4	0.5	
No	613	98.7	3,490	99.3	835	99.5	
Orbital							<0.001 ^a^
Yes	88	14.2	899	25.6	236	28.1	
No	533	85.8	2,616	74.4	603	71.9	
Nasal							<0.001 ^a^
Yes	373	60.1	1,467	41.7	264	31.5	
No	248	39.9	2,048	58.3	575	68.5	
NOE							0.485 ^a^
Yes	0	0	5	0.1	2	0.2	
No	621	100	3,510	99.9	837	99.8	
ZMC							<0.001 ^a^
Yes	64	10.3	727	20.7	258	30.8	
No	557	89.7	2,788	79.3	581	69.2	
Mandible							<0.001 ^a^
Yes	72	11.6	300	8.5	48	5.7	
No	549	88.4	3,215	91.5	791	94.3	
Panfacial							0.614 ^a^
Yes	16	2.6	92	2.6	27	3.2	
No	605	97.4	3,423	97.4	812	96.8	

Abbreviations: ZMC, zygomaticomaxillary complex; NOE, naso-orbito-ethmoidal.

Note: Orbital = orbital floor + other orbital; ZMC = maxillary + ZMC + LeFort.

Chi-square test was used.

**Fig. 4:**
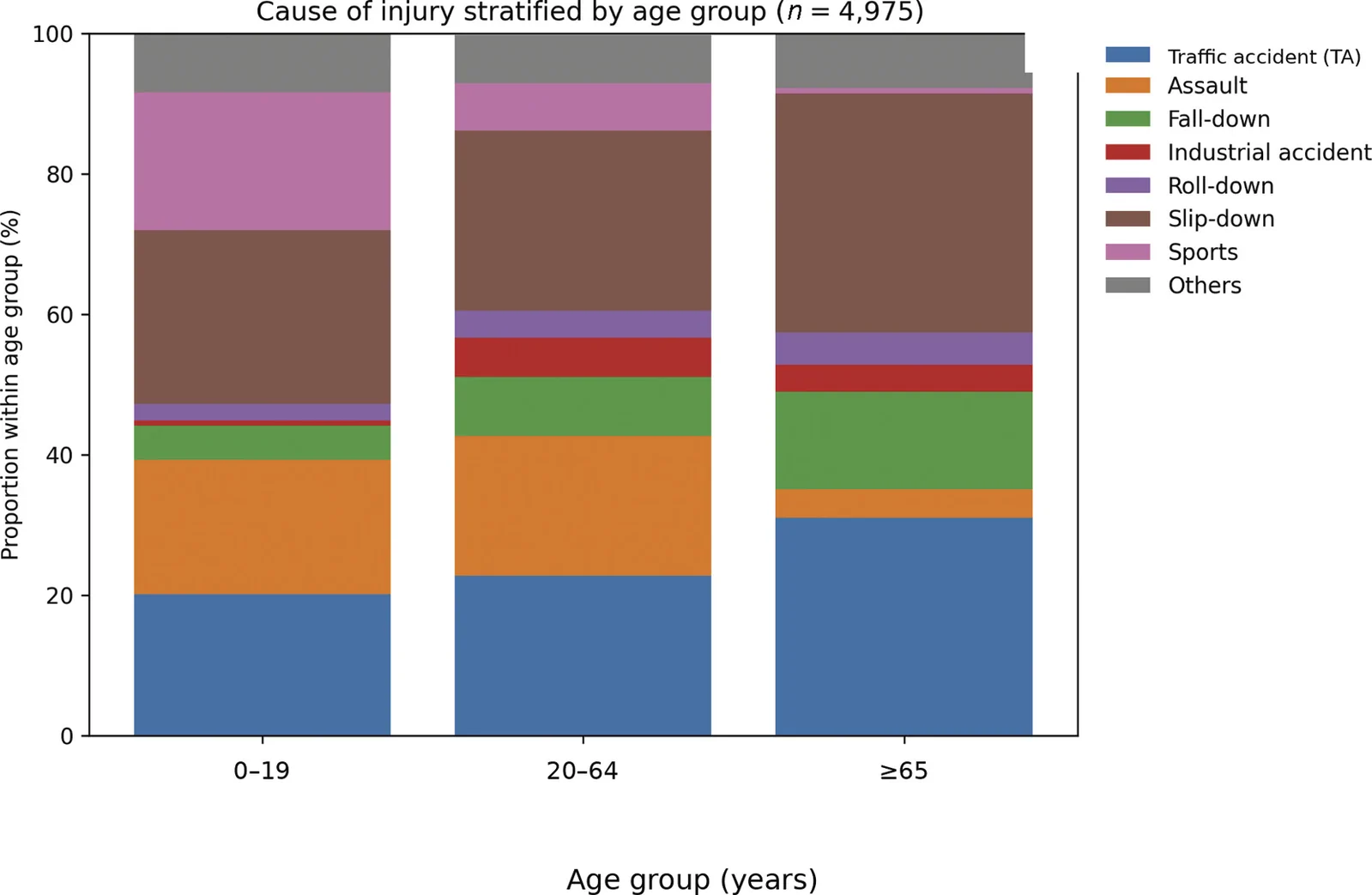
Etiology of facial bone fractures by age groups. Slip-down was the most common cause across all ages and increased in the elderly; traffic accidents also represented a relatively larger share in elderly patients, whereas assault- and sports-related injuries were more frequent in younger patients.

The distribution of injury mechanisms varied significantly across age strata ( Table 3 and Fig. 4 ). Falls (slip-down, roll-down, and fall-down combined) and TAs predominated in elderly patients, whereas assaults and sports-related trauma accounted for higher proportions in younger groups.

**Table 3:** Cause of injury ( *n* = 4,975)

Variable	Pediatric (age <20 years)		Adult (age 20–64 years)		Elderly (age ≥65 years)		*p* -Value
	*N*	Percentage	*N*	Percentage	*N*	Percentage	
	621	12.5	3,515	70.7	839	16.9	
Traffic accident							<0.001 a
Yes	125	20.1	802	22.8	260	31	
No	496	79.9	2,713	77.2	579	69	
Assault							<0.001 a
Yes	119	19.2	701	19.9	34	4.1	
No	502	80.8	2,814	80.1	805	95.9	
Fall-down							<0.001 a
Yes	30	4.8	297	8.4	117	13.9	
No	591	95.2	3,218	91.6	722	86.1	
Industrial accident							<0.001 a
Yes	5	0.8	198	5.6	32	3.8	
No	616	99.2	3,317	94.4	807	96.2	
Roll-down							0.056 a
Yes	14	2.3	135	3.8	39	4.6	
No	607	97.7	3,380	96.2	800	95.4	
Slip-down							<0.001 a
Yes	154	24.8	903	25.7	286	34.1	
No	467	75.2	2,612	74.3	553	65.9	
Sports							<0.001 a
Yes	122	19.6	237	6.7	6	0.7	
No	499	80.4	3,278	93.3	833	99.3	
Others							0.337 a
Yes	52	8.4	242	6.9	65	7.7	
No	569	91.6	3,273	93.1	774	92.3	

a Chi-square test was used.

**Fig. 5:**
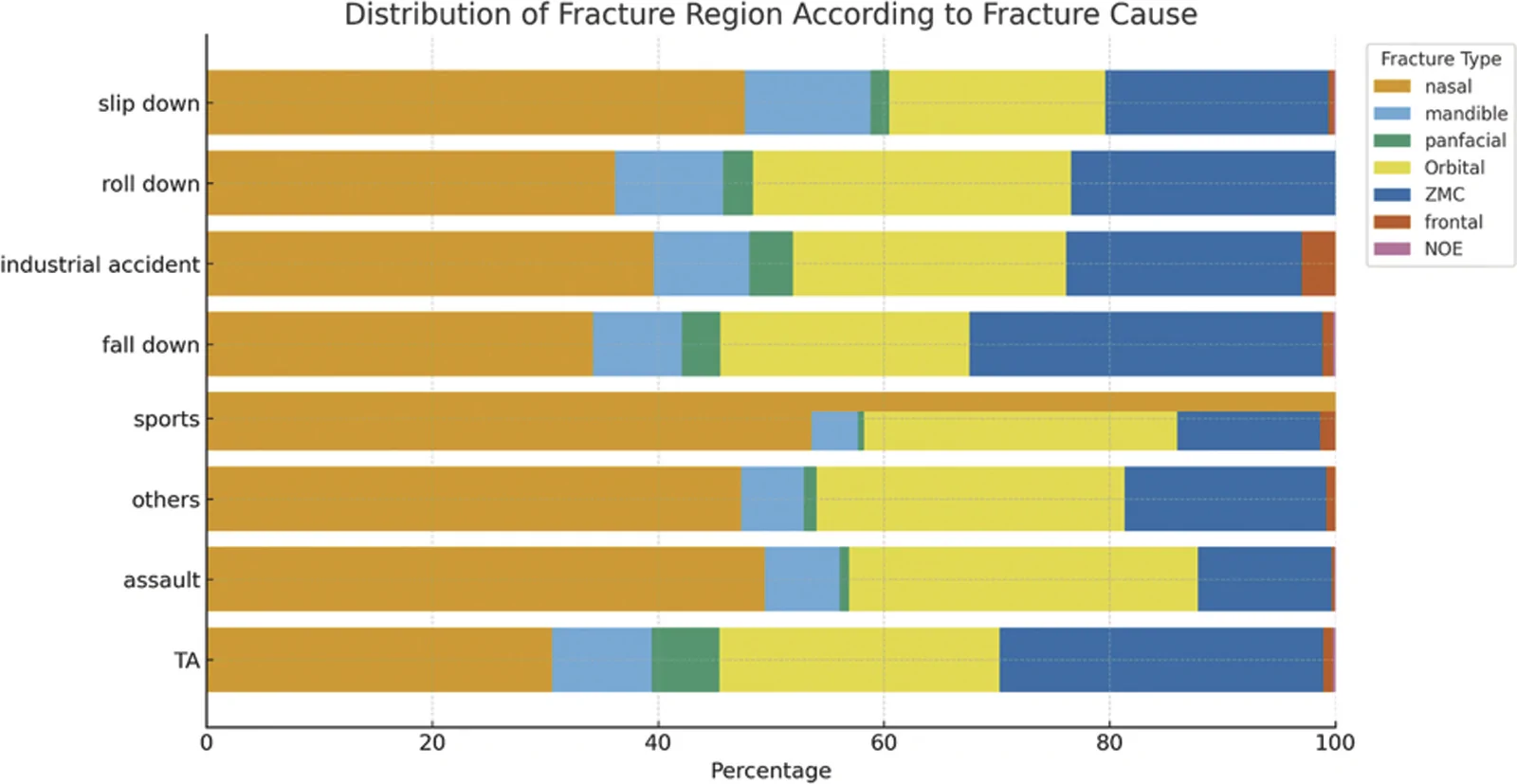
Association between fracture etiology and fracture site. Fracture patterns varied according to injury mechanism. Assaults most frequently involved nasal and orbital wall fractures, reflecting direct anterior blows. Slip-down and fall-down also commonly produce orbital/nasal fractures, but with lower nasal proportions than assault. Traffic accidents and industrial accidents generated a broader spectrum with higher ZMC and mandibular involvement and more complex patterns, while sports injuries were mainly nasal/orbital. NOE, naso-orbito-ethmoidal; ZMC, zygomaticomaxillary complex.

Mechanism of injury was strongly associated with fracture site ( *p* < 0.001): Assaults were enriched for nasal—with some orbital—involvement; falls (slip/roll/fall-down) also produced predominantly nasal/orbital patterns but with lower nasal proportions than assault; traffic-related and industrial accidents showed a broader spectrum with higher ZMC and mandibular proportions; sports injuries were mainly nasal/orbital; panfacial patterns were uncommon across mechanisms ( Fig. 5 ).

In supplementary temporal analyses, weekday case counts were non-uniform (chi-square test, *p* < 0.001): Monday was highest (904), and Saturday was the lowest (488); counts from Tuesday to Friday were 786, 755, 795, and 703, respectively, with 544 on Sunday. Pairwise testing confirmed a weekend effect—most contrasts involving Saturday (and some Sunday) versus weekdays remained significant after Holm-adjusted *p* < 0.05—whereas weekday-to-weekday differences were largely non-significant ( Supplementary Fig. 1A, B [available in the online version only]). Day-of-month counts (1st–10th: 1,593; 11th–20th: 1,676; 21st–31st: 1,706) did not differ significantly, and annual case counts by year are summarized in Supplementary Table 1 (available in the online version only). Annual case counts were comparable between the pre-COVID (2016–2019) and COVID (2020–2021) periods (Poisson rate ratio 0.98, 95% CI, 0.92–1.04; *p* = 0.428), and annual totals showed no consistent trend ( Supplementary Figs. 2 and 3 [available in the online version only]).

To further identify independent predictors of surgical intervention, we performed a multivariable logistic regression analysis (complete-case analysis; *n* = 4,767) including age, sex, osteoporosis, cognitive impairment, and multiple fractures ( Table 4 ). In multivariable analysis, increasing age was independently associated with a lower likelihood of operative management (aOR 0.98 per 1-year increase; 95% CI, 0.977–0.983; *p* < 0.001). In contrast, multiple fractures (two or more distinct anatomical regions) were associated with higher odds of surgery (aOR 2.50; 95% CI, 1.57–3.99; *p* < 0.001). Male sex (aOR 0.94; 95% CI, 0.81–1.08; *p* = 0.356), osteoporosis (aOR 1.96; 95% CI, 0.65–5.92; *p* = 0.232), and cognitive impairment (aOR 0.70; 95% CI, 0.41–1.18; *p* = 0.180) were not independently associated with operative management.

**Table 4:** Multivariable logistic regression for operative management among patients with facial bone fractures (complete-case analysis; *n* = 4,767)

Variable	Adjusted OR	95% CI	*p* -Value
Age (per 1-year increase)	0.98	0.977–0.983	<0.001
Male sex	0.94	0.81–1.08	0.356
Osteoporosis	1.96	0.65–5.92	0.232
Cognitive impairment	0.70	0.41–1.18	0.180
Multiple fractures (two or more distinct anatomical regions)	2.50	1.57–3.99	<0.001

Abbreviations: CI, confidence interval; OR, odds ratio.

Note: Outcome was operative management (yes = 1) versus conservative management (no = 0). Multiple fractures were defined as fractures involving two or more distinct anatomical regions (or two or more fracture-site categories) within a single injury episode.

Values are presented as adjusted odds ratios with 95% confidence intervals.

## Discussion

This large, single-center cohort identified clear age-related differences in the epidemiology and management of facial fractures across the lifespan. By examining age-stratified prevalence and clinical characteristics of facial bone fractures in 4,975 patients, we sought to deepen the understanding of facial trauma across different age groups and to provide data that may assist in developing age-appropriate prevention measures and treatment planning. As a designated regional trauma center, our institution captures a broad case-mix, and the cohort size compares favorably with prior single-center series, enabling robust characterization of age-specific patterns from pediatric to elderly patients. While many studies describe facial-fracture epidemiology within specific adult or geriatric cohorts, direct, within-dataset comparisons across multiple age strata remain relatively uncommon; our cohort addresses this gap by spanning pediatric, working-age adult, and elderly patients. 2 3 11 Moreover, although nasal fractures consistently dominate overall counts, prior literature has incompletely characterized how the relative proportions of ZMC, orbital, and mandibular fractures vary by age; our data delineate these differences with explicit percentages across strata. 5 6 11

Although nasal fractures were most frequent overall, their proportion declined with age, whereas ZMC and orbital fractures increased among elderly patients. 2 3 8 Conversely, mandibular fractures were more common in younger individuals, paralleling exposure to high-energy mechanisms such as TAs and assaults. 1 6 7 These patterns align with multicenter and national data indicating that trauma energy and bone quality are major determinants of fracture configuration. 3 4 9 11

The predominance of nasal bone fractures in this and other series likely reflects both anatomical vulnerability and detection-related factors. The nasal bones form the most anterior and centrally projected portion of the facial skeleton, are relatively thin, and are only modestly supported by adjacent buttresses, making them highly susceptible to even moderate blunt impacts to the midface. 2 5 6 Common mechanisms in our cohort—such as interpersonal violence, sports-related collisions, ground-level falls, and road TAs—frequently involve force vectors directed toward the nasal dorsum rather than the deeper midfacial buttresses, predisposing to isolated nasal injuries. 7 8 14 In addition, external deformity, swelling, and epistaxis are readily recognized by patients and caregivers, which may increase the likelihood of emergency department presentation and CT imaging compared with subtler orbital or ZMC fractures. Together, these anatomical and clinical factors provide a plausible explanation for the high proportion of nasal fractures observed in our hospital-based cohort and in previous epidemiologic reports. 2 3 5 11

Mechanisms of injury in our study showed clear age-dependent patterns that help to contextualize these fracture distributions. Among elderly patients, slip-down, TAs, and fall-down were the leading causes (34.1%, 31.0%, and 13.9%, respectively), and when slip-, roll-, and fall-down were combined, falls accounted for more than half of elderly facial fractures (52.6%). Assault and sports-related injuries =were comparatively rare in this group (4.1% and 0.7%), consistent with reduced physical activity and social exposure. In contrast, pediatric patients demonstrated a more mixed profile, with slip-down (24.8%), sports (19.6%), assault (19.2%), and TAs (20.1%) each contributing substantially, reflecting high levels of play- and school-related activity as well as interpersonal violence. Adults showed an intermediate pattern, in which TAs (22.8%), slip-down (25.7%), and assault (19.9%) together accounted for the majority of injuries, alongside a higher proportion of industrial accidents compared with the other age groups.

These age-specific differences in mechanism and activity level are closely linked to fracture-site distribution. More broadly, the predominant mechanisms of facial trauma are not identical across countries and health care systems. Regional differences in transportation habits, occupational exposure, alcohol use, interpersonal violence, and population age structure can substantially influence both injury etiology and fracture distribution. For example, in countries with high cycling prevalence, bicycle- and e-bike-related injuries may account for a substantial proportion of facial trauma. 15 Therefore, our findings should be interpreted as reflecting the epidemiologic context of a regional trauma center in South Korea rather than a universally uniform distribution. Among pediatric patients, nasal fractures predominated (60.1%), whereas ZMC fractures were comparatively infrequent (10.3%) and mandibular fractures were more common than in the elderly (11.6% vs. 5.7%). This distribution mirrors the mixed, play- and school-related mechanisms observed in this group; in clinical decision-making, radiation minimization and growth-related considerations remain relevant, although operative care may be required for displaced injuries. 6 7 11 Elderly patients, who frequently sustain ground-level or low-height falls, often experience lateral or oblique impact to the malar region; coupled with diminished protective reflexes and age-related cortical thinning, this may predispose to ZMC and orbital fractures even when absolute energy transfer is modest. 8 10 Younger individuals, particularly pediatric and working-age adults, are more often exposed to high-energy events such as TAs, contact sports, and assaults, which generate complex loading across the mandible and midfacial buttresses and thereby increase the likelihood of mandibular and combined midfacial injuries. 1 6 7 Consistent with this, our cause-by-site analysis showed that assaults and sports injuries were enriched for nasal and orbital involvement, whereas traffic and industrial accidents produced a broader spectrum of fracture configurations with higher proportions of ZMC, mandibular, and panfacial patterns. The relatively higher ZMC fraction in elderly patients may also reflect the case mix of a tertiary trauma-center cohort, in which minor isolated nasal fractures—particularly in younger patients—are often treated conservatively in outpatient settings without CT confirmation, leading to a relative enrichment of more complex midfacial injuries in institutional registries. 2 5 Among elderly patients sustaining facial trauma, soft tissue injuries commonly exceed fracture diagnoses; thus, our fracture-only registry reflects a more severe spectrum of elderly presentations and may consequently accentuate the proportion of midfacial fractures. 14 16 17 18

Our supplementary temporal analyses demonstrated a clear weekday-skewed pattern in case counts, with the highest volume on Monday and the lowest on Saturday, indicating a pronounced weekend effect. In contrast, we observed no significant differences across day-of-month sections and no consistent year-to-year trend; additionally, the COVID-19 period was not associated with a meaningful change in overall case volume. Collectively, these findings suggest that facial trauma presentations may be more closely linked to routine social activity structures—particularly weekday commuting, occupational exposure, and school-related activities—than to seasonal variation, annual fluctuations, or transient societal events. The weekday-to-weekday differences were modest, whereas the reduction during the weekend was distinct, supporting the possibility that a substantial proportion of cases are related to work- or school-based activities and commuting-related exposures. The observed Monday peak may reflect increased exposure as routine activities resume after the weekend, as well as potential influences related to care-seeking timing; however, these explanations remain speculative. Clinically, a weekday-predominant demand pattern may inform resource allocation for facial trauma care (e.g., staffing and operating room readiness), while prevention efforts may prioritize commuting and occupational safety alongside age-tailored fall and violence prevention. Nevertheless, because this was a retrospective study and contextual exposure data were unavailable, the underlying drivers of these temporal distributions cannot be determined. In addition, temporal distributions are likely to vary across countries and social settings according to the predominant mechanisms of injury. In regions where interpersonal violence-related trauma accounts for a larger proportion of facial injuries, incidence may instead peak from Friday to Sunday. 19 Therefore, the weekday-skewed pattern observed in our cohort should be interpreted as context-dependent rather than universal.

Age also influenced treatment decisions. Operative management was less frequent in elderly patients, whereas conservative treatment predominated in this group. Importantly, the lower operative rate observed in elderly patients likely reflects differences in surgical candidacy (frailty, comorbidity burden, and anesthesia risk) and functional priorities, rather than chronological age per se. Our multivariable analysis extends these observations by demonstrating that advancing age is an independent negative predictor for surgical intervention, even after adjusting for sex and recorded comorbidities. This finding suggests that the physiological burden of aging and potentially higher perioperative risks significantly influence the choice of conservative management in the elderly. Although multiple fractures were uncommon (165/4,975 cases, 3.3%), corresponding to 81 unique patients (1.7%), our multivariable regression analysis identified them as the strongest independent predictor for surgical intervention (aOR = 2.50, *p* < 0.001). This highlights that while systemic factors (age) may deter surgery, the severity and complexity of the injury remain the primary clinical indicators for operative reconstruction. These results support a nuanced approach to surgical decision-making that balances the anatomical necessity for repair against the patient's overall frailty and functional priorities.

Contemporary guidance for trauma in elderly patients emphasizes individualized decision-making that weighs fracture pattern, comorbidities, frailty, and overall functional status; in many cases, conservative treatment is appropriate. 20 21 Functional outcomes (speech, mastication, ocular alignment/vision) and cognitive recovery should take priority over aesthetic restoration when trade-offs are necessary. 8 20 21 Given immune senescence and impaired tissue regeneration, meticulous infection prevention and early recognition of tissue necrosis are critical, and early multidisciplinary rehabilitation—including nutrition optimization—supports recovery and independence. 8 20 21

In addition to age, comorbidity, and mechanism, formal injury-severity scoring systems may further assist risk stratification in facial trauma. The Facial Injury Severity Scale (FISS) offers a simple anatomy-based estimate of maxillofacial injury burden, 22 whereas the Abbreviated Injury Scale (AIS) is widely used in trauma systems to grade anatomical injury severity and support overall polytrauma assessment. 23 The Comprehensive Facial Injury (CFI) score has also been proposed as a practical facial trauma severity tool with reported associations with operative burden and hospitalization requirements. 24 25 Although these scores were not available for standardized retrospective reconstruction in our registry, future age-stratified studies incorporating severity scores may better predict operative time, length of hospital stay, associated injuries, and complication risk.

Time from injury to surgery and hospital length of stay tended to be longer with advancing age and comorbidity in our cohort, likely reflecting preoperative optimization and extended postoperative monitoring. This pattern is consistent with previous literature emphasizing comorbidity burden, anticoagulant and antiplatelet use, anesthetic risk, and functional priorities in elderly trauma patients, 9 10 20 26 27 as well as an increased risk of associated injuries in elderly facial fracture cohorts. 28 These observations support individualized algorithms that balance anticipated functional and aesthetic benefit against systemic risk, particularly in frail or polypharmacy populations.

From a preventive standpoint, the predominance of fall-related fractures in elderly patients underscores the value of comprehensive fall-prevention bundles—balance and strength training, home hazard mitigation, vision and footwear review, and medication reconciliation, especially regarding sedatives and antihypertensives. 4 8 29 In contrast, strategies for younger cohorts should focus on high-energy injuries, including road traffic safety (seatbelts, helmets, and impaired driving prevention), sports safety measures, and violence reduction initiatives. 6 7 11 For pediatric patients, prevention should address school and playground environments, appropriate supervision, and early counseling regarding risk-taking and interpersonal violence.

A distinct strength of this study is the high reliability of the dataset. Rather than relying solely on administrative diagnostic codes, which can be inaccurate, we manually verified all CT images to confirm the exact fracture sites and patterns. This rigorous validation process ensures that our analysis of age-related fracture distribution and surgical decision-making is based on confirmed anatomical data, minimizing the risk of misclassification bias inherent in large-scale registry studies.

This study has limitations. First, this study was conducted at a single regional trauma center in Gangwon Province, South Korea, and the cohort consisted almost exclusively of Korean patients. Consequently, the observed injury mechanisms and fracture patterns may reflect specific regional, geographic, or cultural characteristics, which may limit the generalizability of our findings to other ethnic groups or populations with different trauma systems. Additionally, as a hospital-based study, it may underrepresent minor non-operative injuries (e.g., simple nasal fractures managed on an outpatient basis). Second, radiologic granularity was partly aggregated. Although orbital wall fractures were captured as floor and other walls (medial, lateral, and roof) at data entry, these were reported as a single “orbital” category in the main comparative analyses and figures to reduce sparsity; Le Fort subtypes and ZMC severity were likewise not stratified. Third, fracture severity (displacement and comminution), laterality, and quantitative energy metrics were not systematically captured; standardized CT-based severity scales (e.g., Zingg classification and Le Fort subclassification) and interrater reliability for site coding were not assessed. Fourth, treatment selection (operative vs. conservative) is subject to surgeon preference and patient comorbidity, introducing confounding that is not fully eliminated by group comparisons. In addition, comorbidities were abstracted from retrospective medical records and may be under-recorded, which could attenuate observed between-group differences. Fifth, outcomes beyond the index hospitalization (occlusion, diplopia or enophthalmos, infection, reoperation, and readmission) were not evaluated, precluding linkage of fracture pattern and treatment modality to functional results. Sixth, although we described basic temporal patterns and compared annual case counts between the pre-COVID-19 and COVID-19 periods, we did not formally model seasonal variation or pandemic-related policy effects on incidence. Collectively, these constraints may bias site and mechanism proportions toward more complex, admitted cases. Nevertheless, with nearly 5,000 patients spanning pediatric to elderly groups, the present analysis provides a robust, age-stratified overview that complements existing multicenter and national data in a rapidly aging society. 12

### Conclusion

This study demonstrated distinct age-dependent patterns of facial bone fractures. Children most frequently sustained nasal fractures with mixed, relatively lower-energy mechanisms, working-age adults showed higher proportions of mandibular and ZMC fractures associated with higher-energy trauma, and elderly patients exhibited relatively higher proportions of ZMC and orbital fractures, with falls representing the predominant mechanism. Understanding these age-specific patterns may help inform tailored prevention strategies and individualized treatment planning across the lifespan.

## Declaration of GenAI Use

Generative artificial intelligence (ChatGPT; OpenAI) was used to assist with language editing and improvement of manuscript clarity. All content was reviewed and verified by the authors who take full responsibility for the integrity and accuracy of the manuscript.
