# Supplementary material for: Epidemiological and Clinical Characteristics of Facial Bone Fractures across Age Groups: A Single-Center Retrospective Analysis of 4,975 Patients at a Regional Trauma Center in Gangwon Province, South Korea
Source: Arch Plast Surg. 2026 Aug 11;53(4):331–41. doi: 10.1055/a-2870-8157 (PMC13496214; doi:10.1055/a-2870-8157)
Supplement: Supplementary file 1 [file 10-1055-a-2870-8157_29283376.pdf]

Supplementary Fig. 1 (**A**). Weekly case counts of facial bone fractures by day of week (2016–2021; n=4,975). Bars show the number of patients per weekday with value labels above each bar (Monday 904; Tuesday 786; Wednesday 755; Thursday 795; Friday 703; Saturday 488; Sunday 544). The distribution was non-uniform (chi-square test, $p < 0.001$); weekdays (Mon–Fri) accounted for 3,943 (79.3%) versus weekend (Sat–Sun) 1,032 (20.7%). (**B**). Pairwise weekday comparisons of incidence. Heatmap of pairwise weekday contrasts with color intensity proportional to $-\log_{10}$(p-value); asterisks mark comparisons that remained significant after Holm adjustment (two-sided, $p < 0.05$). The strongest differences involved Saturday (and some Sunday) versus weekdays, whereas most weekday-to-weekday contrasts were not significant.

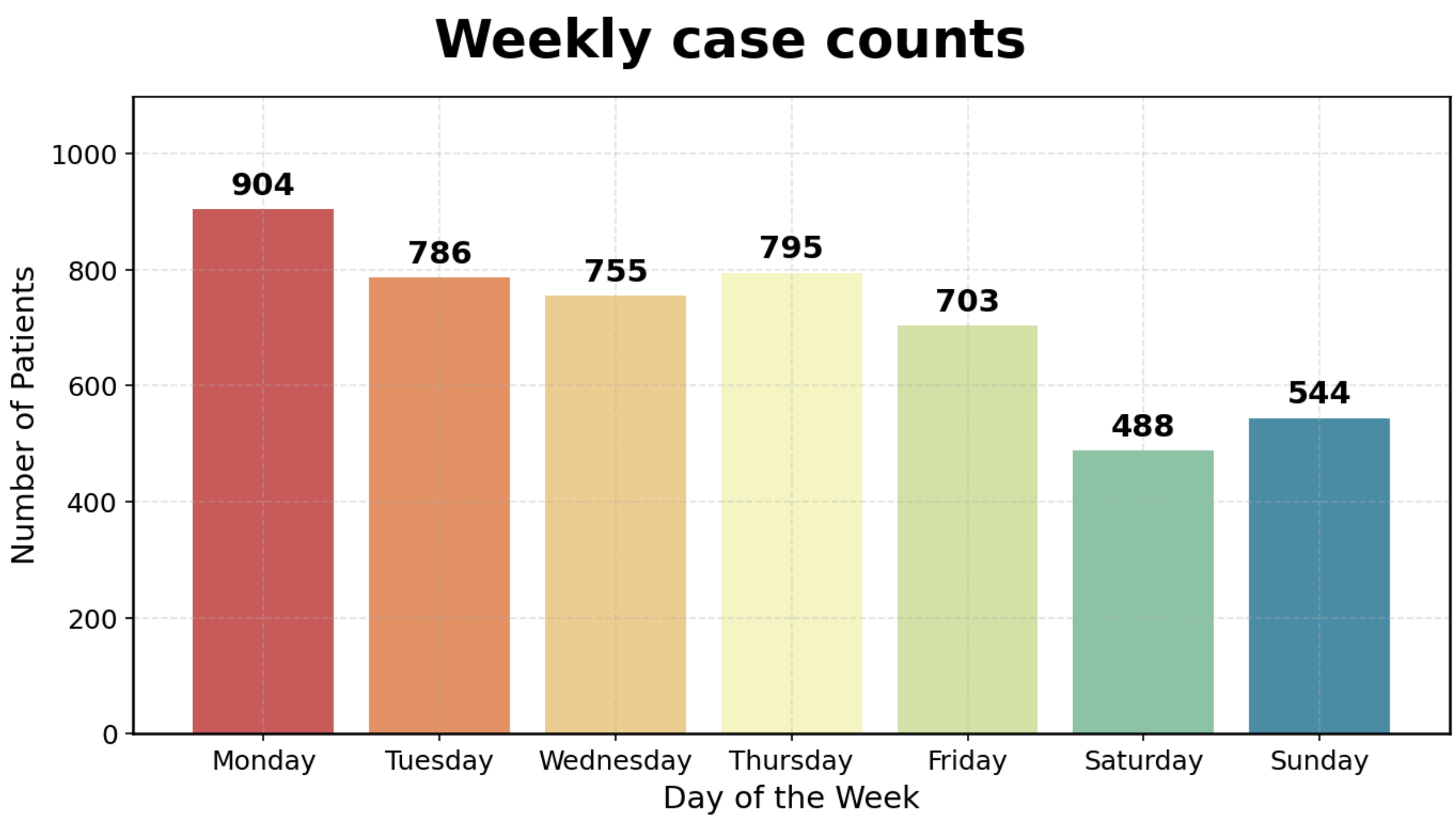

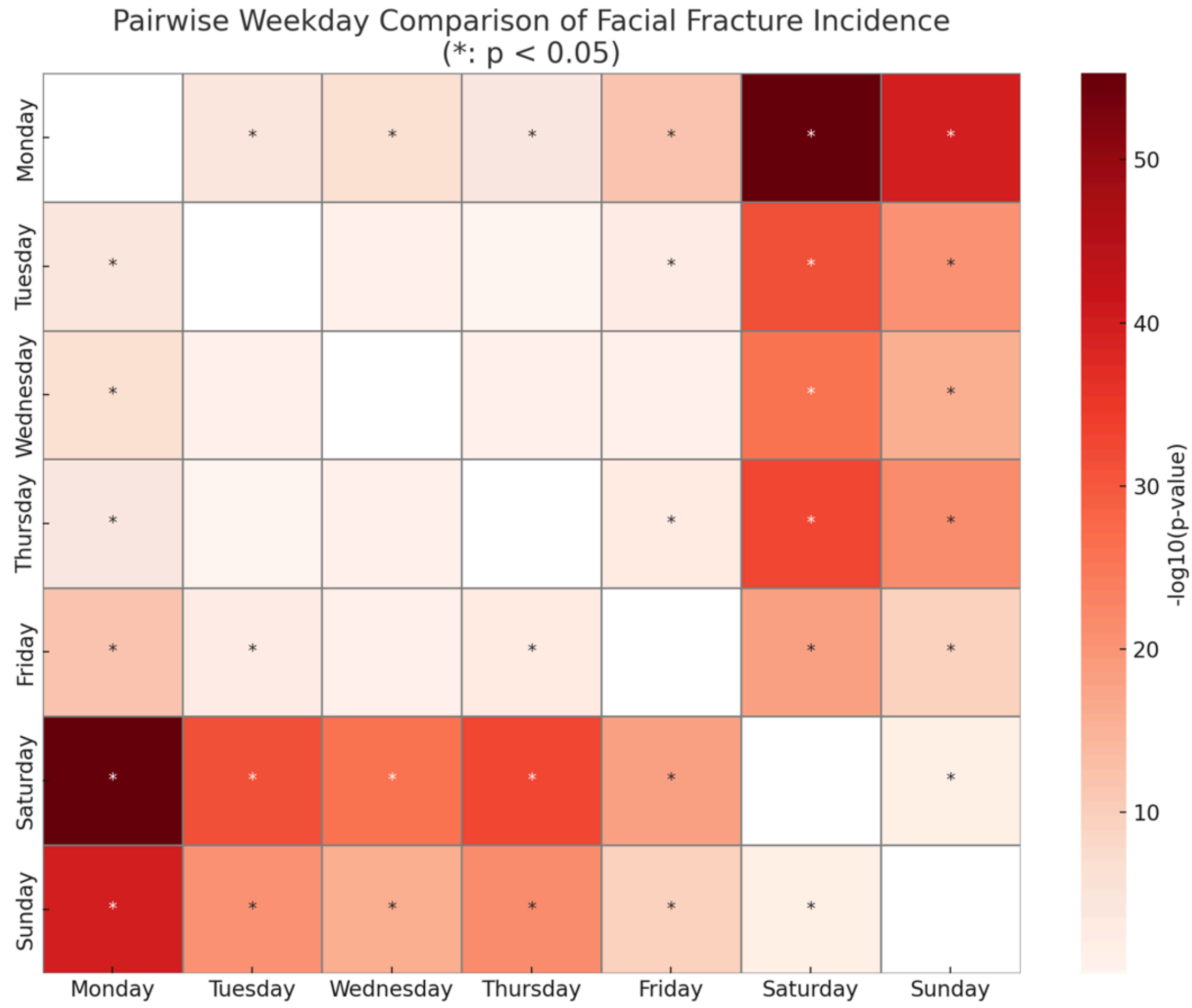

Supplementary Fig. S2 Distribution of facial bone fractures by day of month (1st–10th: 1,593; 11th–20th: 1,676; 21st–31st: 1,706). No significant differences across segments.

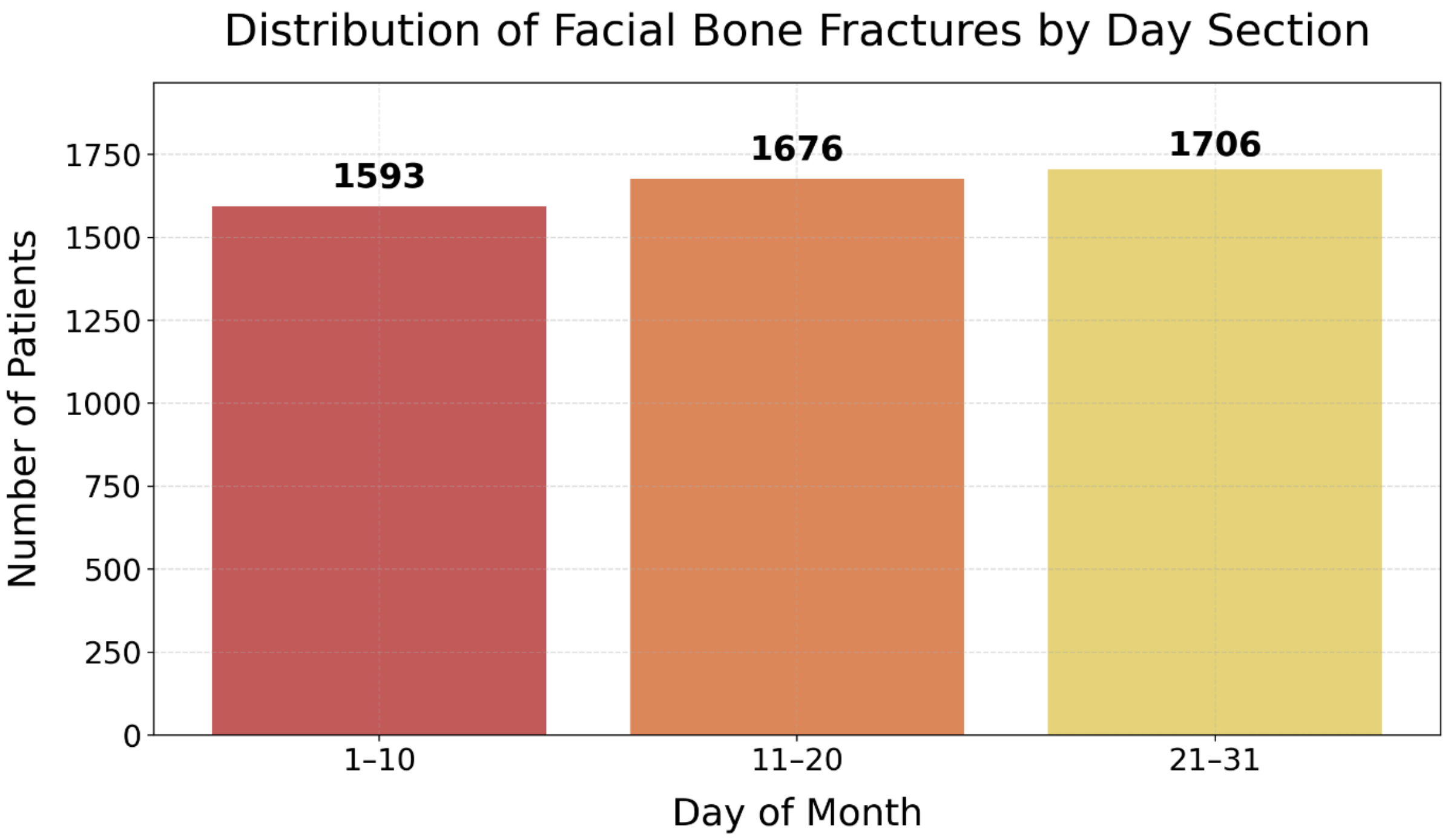

Supplementary Fig. S3 Annual case counts (2016–2021) with value labels; no consistent temporal trend.

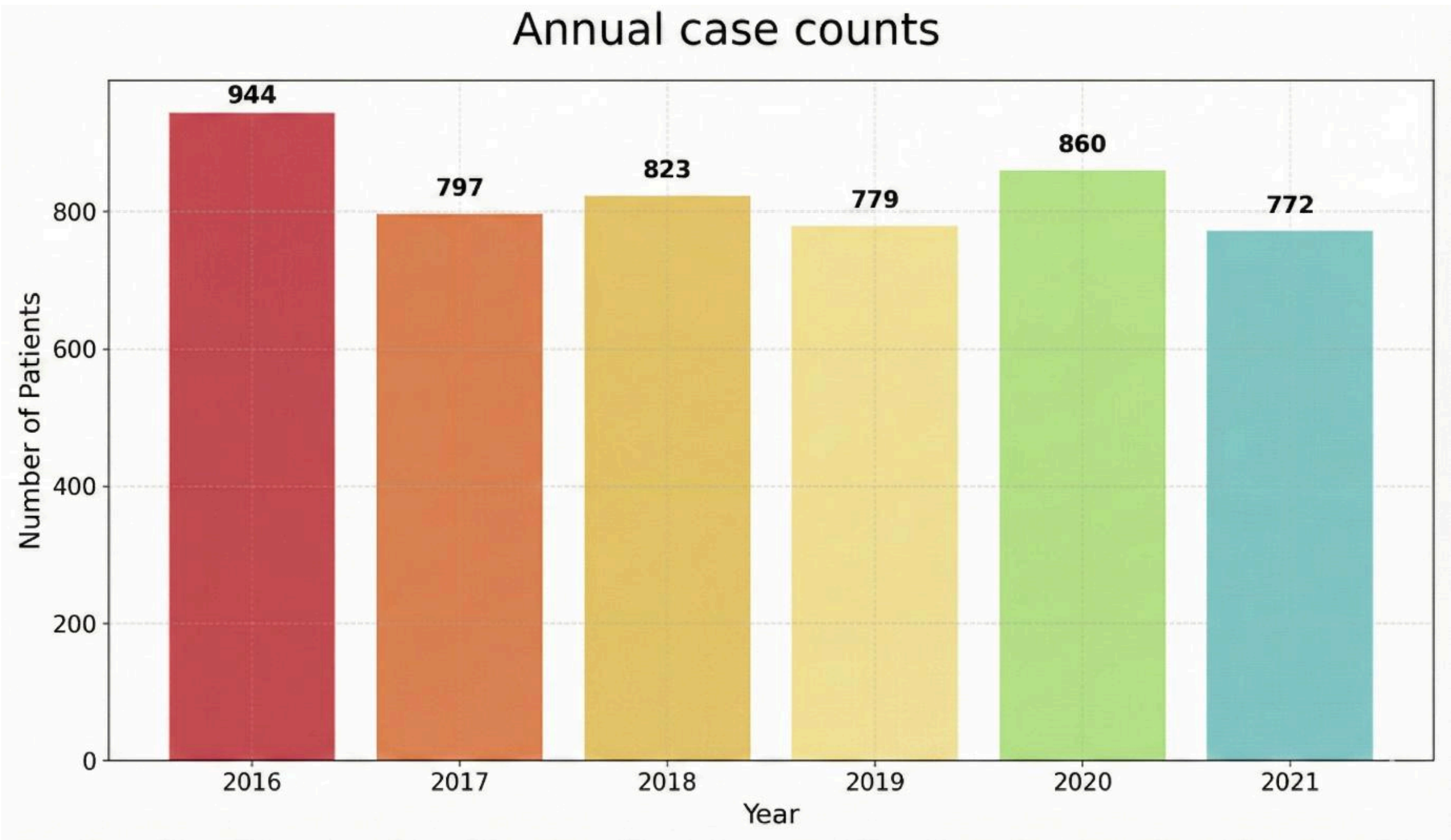

Supplementary Table 1. Annual number of enrolled patients by year (2016-2021)

Year	No. of patients (n, %)
**2016**	**944 (19.0%)**
**2017**	**797 (16.0%)**
**2018**	**823 (16.5%)**
**2019**	**779 (15.7%)**
**2020**	**860 (17.3%)**
**2021**	**772 (15.5%)**
**Total**	**4975 (100.0%)**

Note. Values are presented as *n (%)*. The annual number of enrolled patients did not differ significantly between the pre–COVID-19 (2016–2019) and COVID-19 (2020–2021) periods (incidence rate ratio [IRR], 0.98; 95% CI, 0.92–1.04; p=0.428; Poisson rate ratio test).
Abbreviations: CI, confidence interval.
